# Integrating Occupational Therapy Specific Assessments in Practice: Exploring Practitioner Experiences

**DOI:** 10.1155/2017/7602805

**Published:** 2017-12-03

**Authors:** Eric Asaba, Mio Nakamura, Akie Asaba, Anders Kottorp

**Affiliations:** ^1^Department of Neurobiology, Care Sciences, and Society (NVS), Division of Occupational Therapy, Karolinska Institutet, Alfred Nobels Allé 23, B4, Huddinge, Sweden; ^2^Unit for Research, Education, and Development (FoUU), Stockholms Sjukhem Foundation, Stockholm, Sweden; ^3^Department of Occupational Therapy, Tokyo Metropolitan University, Tokyo, Japan; ^4^Department of Occupational Therapy, International University of Health and Welfare, Narita, Japan; ^5^Karolinska University Hospital, Occupational Therapy and Physiotherapy Practice Areas, Huddinge, Sweden; ^6^Faculty for Health and Society, Malmö University, Malmö, Sweden

## Abstract

**Background:**

Occupational therapists sometimes find it challenging to integrate client-centered and occupational therapy specific assessments in practice.* The aim* of this study was to explore the use of occupational therapy specific assessments such as the Assessment of Motor and Process Skills (AMPS) among occupational therapists in Sweden and Japan.

**Methods:**

Interviews and qualitative thematic analyses were utilized.

**Findings:**

Four themes are reported: (1) use it or lose it, (2) simply no space until after hours, (3) biggest barriers can be colleagues, and (4) being more specific: communication.

**Conclusion:**

In keeping with previous studies, occupational therapists often find it challenging to implement client-centered and occupation-based assessment tools into practice. However, more work is needed to understand how best practices can be incorporated into a changing occupational therapy daily practice.

## 1. Introduction

Occupational therapists have during many decades used a wide range of formalized assessment tools, including tools borrowed from other disciplines, in order to provide relevant services to clients. During the last 20 years, the profession has seen a steady increase in the development and validation of assessment tools that more distinctly reflect occupational therapy domains of practice such as quality of occupational performance [1, 2], occupational gaps [3], and quality of social interaction [4] to name a few. Occupational therapists use assessment tools with an intended purpose to better guide intervention planning and to provide baseline and outcome measures in order to track progress and/or change among clients. For the purposes of this study, the authors adopt Fisher's [5] differentiation between occupation-centered, occupation-based, and occupation-focused assessments. Occupation-centered assessment refers to the centrality of occupation in informing decisions and action, a perspective; occupation-based assessment refers to situations in which occupation is a fundamental ingredient. Occupation-focused assessment refers to keeping the focus on occupation, with an immediate impact on occupational performance. For instance, an assessment in which occupation is performed as an essential part would by this definition be occupation-based, while an assessment in which goals pertaining to occupational performance are discussed and set, but where the particular occupations are not performed, would be occupation-focused.

## 2. Background

In a study conducted in northern Sweden, occupational therapists working in an acute hospital facility were interviewed regarding their experiences with integrating client-centeredness and occupation-focus into their practice based on the use of the Occupational Therapy Intervention and Process Model (OTIPM) [6]. Unsurprisingly, occupational therapists experienced a transformation of thought and action as they explicitly worked with a model to critically reflect on their own practices. Their experiences were imbued with both relief from the increased clarity with which they were working and ambiguity from hinders in the healthcare systems and the difficulties in changing organizational culture.

Contrary to the above depiction of deeply engaged therapists spending substantial time in continuing education and reflecting on their practice, it has also been documented that occupational therapists might find it challenging to incorporate specific tools in practice. In one study, 260 occupational therapists were surveyed, finding that occupational therapists most often choose an assessment tool because of availability and ease of administering the assessment (including ease of scoring and time efficiency) or that the tool facilitated client-centeredness [7]. In another study utilizing a survey and follow-up interviews, 50 therapists who had taken an AMPS course in England were asked about incorporating a new assessment in practice. [8]. Barriers to incorporating new assessment skills in clinical practice included the following: lack of support at the workplace, difficulty selecting appropriate clients, time to complete the assessment, and difficulties getting started. It was concluded that the successful implementation of an assessment tool such as AMPS in clinical practice would hinge upon receiving support from managers in the form of time, space for reflection, and invested resources in a continuum of learning [8]. These studies suggest that the utility of occupational therapy assessments might be explained by availability and ease of incorporation in practice rather than the soundness of the psychometric qualities of the assessment [7, 8].

Although client-centeredness and occupation are not new to occupational therapy, occupational therapists still find it difficult to incorporate client-centered and occupation-based or occupation-focused assessments in practice [7–10], even though occupational therapists place value on this type of assessment when asked. It is of importance to study the potential gap between practices in which occupation for different reasons cannot be integrated and a vision in which the value of occupation is central.

It is also relevant from an international perspective. A critique to the importation/exportation of theories (and implicitly assessments grounded in such theories) from one country context to another is well known [11]. It continues to be relevant and important to explore how occupational therapists reflect on their practice [12, 13] in order to situate clinical reasoning in different country/practice settings. Because the utility of assessments from a country/practice perspective is constantly evolving, it is of relevance to periodically and systematically explore experiences of using assessments among occupational therapists in different country/practice contexts.

The aim of this study is to explore the use of occupational therapy specific assessments such as the Assessment of Motor and Process Skills (AMPS) among occupational therapists working in Sweden and Japan.

## 3. Methods

### 3.1. Design

This study was based on qualitative interviews with occupational therapists from Sweden and Japan who had received training in the Assessment of Motor and Process Skills (AMPS) as well as other occupational therapy specific instruments such as A3 [14]. Data were analyzed in accordance with qualitative thematic analysis [15, 16]. All participants gave informed consent prior to being interviewed. The local ethics committee approved the study.

### 3.2. Participants

Information about the study was sent to occupational therapists in the A3 course database. The participants (14 women and 5 men) in this study expressed interest by making contact through email or telephone (see Table 1 for overview). Between the years 2004 and 2014, the A3 (formerly AAD) course was held 7 times with a total of 81 course participants (39 men and 42 women) in Japan and 8 times in Sweden with a total of 85 course participants (2 men and 83 women). A3, like the AMPS, requires each occupational therapist to submit 10 cases for calibration upon completion of the course. The validity of the instrument in Japanese and Swedish contexts as well as studies incorporating the assessment has been reported elsewhere [14, 17–23]. Recruitment from the A3 database was seen as relevant because all therapists would have also attended an AMPS course and would have recently taken an initiative to update their knowledge by having attended an A3 course.

All participants were licensed to practice occupational therapy in Japan or Sweden. In addition, all Japanese participants were calibrated AMPS raters. The participants in Japan had between 1 and 7 years of experience using AMPS (mean = 3; SD = 1.95) and participants in Sweden had 4–15 years of experience using AMPS (mean = 9; SD = 3.53). Among the participants, there were both those who used assessments on a regular basis and those who did not. Participant practice areas included the following: physical rehabilitation, home rehabilitation, psychiatry, and education. In order to uphold confidentiality, participant demographics are given in aggregate.

### 3.3. Data

Data gathering and analysis were iterative. The two country contexts represented geographic areas where there has been a strong interest in AMPS. This was relevant because by identifying challenges and support in integrating assessments such as these in different contexts new knowledge is facilitated in terms of strategies for integrating critical clinical reasoning in practice across different contexts. Moreover, this type of exploration has not previously been conducted over national borders or in Japan and Sweden.

Focus group interviews were initially used to build on the collective experiences of the participants. The method allowed members within the group to build on or refute experiences based on their own practice. However, due to the challenge of finding a common time and place, individual interviews were added to include perspectives that might otherwise be lost. It was possible to probe with questions in both the individual interviews and focus groups in order to integrate and deepen the information gathered.

Semistructured interview questions in conjunction with follow-up questions following general principles of qualitative research were used to gain an understanding of participant experiences [24]. All participants were interviewed on one occasion at a place of their convenience, which included their workplace, a seminar room at a university campus, a conference venue seminar room, or a café. Questions included the following: “can you tell me about how you use client-centered assessments in your practice,” “what do you experience as challenges using these assessments,” “what has made it possible for you to use these assessments,” and “can you describe how these assessments help you in your practice?” The interviewer sometimes used more direct probes such as “can you describe in more detail?” or, in the case of a group interview, “has anyone else shared this experience?” In this way, the interviews were structured to develop richness around particular topics generated by both interviewer and participants. Interviews lasted between 45 and 90 minutes and were digitally recorded and transcribed. All interviews were performed by the first author or a research assistant in the project.

### 3.4. Data Analysis

The data analysis was based on techniques using thematic analysis [16, 25] and grounded in concepts of analyzing verbal narratives [15]. In keeping with thematic analytic, a back-and-forth process between interviewing and analysis was performed. All audio materials were transcribed near verbatim, retaining pauses, laughter, and nuances of expressions through bracketed qualifiers. These transcripts were carefully read and coded in three steps. All quotations used in this article were translated by the coauthors and the original Japanese text was maintained during the course of the analysis. The software program, Atlas.ti [26], was used during the analysis phase to code and organize data.

The data was analyzed inductively and although this process involved overlapping between steps, the procedure is outlined stepwise here for the purposes of clarity. All Swedish data were independently coded by the first author and all Japanese data were independently coded by the second author. The first author independently coded some of the Japanese data, which served as a way for the first and second authors to discuss and calibrate the coding process. During the analysis, all data were maintained in the original language. The first and second authors had regular meetings to compare and discuss coding during this phase. All data material was finally reviewed by the first author. The translation of quotes was performed by the first, second, and third authors and compared for accuracy. All authors were part of a discussion and interpretations of the findings.

In the first step, open coding entailed identifying and breaking the text into manageable bits that were assigned codes. Codes captured phenomena in one or a few words and were later applied to more segments of the text. Examples of early codes were “no space,” “difficult to explain,” and “don't have time” to name a few. In the second step, codes were merged and previously coded transcripts were revisited; existing as well as new codes within and across participants were sought by comparison across the data. In this way, both open coding and focused coding were used. Codes such as “don't have time” and “it takes time to prepare” were merged and codes such as “changing the schedule” and “evaluations after hours” contributed to a broader abstraction of the data. In the third step, similar codes were consolidated and organized into coherent groupings, whereby 4 themes were identified. These themes represent possibilities and barriers in using client-centered and occupational therapy specific assessments in practice.

## 4. Findings

Findings from the thematic analysis are presented in four themes. The title for each theme is taken from participant quotes under each theme. Although the naming of each theme is intended to be representative, each theme encompasses more than the phrase: (1) use it or lose it, (2) simply no space until after hours, (3) biggest barriers can be the work culture, and (4) being more specific: communication. The occupational therapists interviewed in this study unanimously communicated an interest in informing their practice with client-centeredness and occupation-focused and/or or occupation-based assessments and interventions. Yet obstacles were also described. Barriers were consistent for occupational therapists in both Swedish and Japanese contexts; however, it is also important to note that some participants in both contexts did not experience these problems. Finally, an interesting finding was that several participants, when pushed to reflect on what they could do to change their situation, reflected that they probably could incorporate more client-centeredness and occupation-based and occupation-focused practice into their work.

## 5. Use It or Lose It

Something that influenced the choice in what assessments to use was the participant's own familiarity with the assessment as well as the extent to which a particular assessment was situated as routine within their practice. One participant said the following:

I simply think, with regard to evaluations, if you don't use it you loose [sic] it and you just don't get it.

Along the same lines, another participant expressed the following:

But often I experience that it is a bit slow in the beginning, but then the more I do the assessments the faster I get…and it takes time to sit with the AMPS bible, one has to look things up, and learn things, if one should put a 4 or a 2 or a 1 for each item…but it takes time, but then when one masters it, then it doesn't take as long.

All the participants used various assessments in practice but were here referring to occupational therapy specific assessments such as AMPS, A3, and COPM. According to these participants, these occupational therapy specific assessments often required more time and effort to perform than using hospital or facility specific ADL or goal checklists. On the other hand, participants were motivated to use the occupational therapy specific assessments because they felt that using these assessments yielded relevant information and made intervention planning clearer. Another participant says the following:

If one does many, it is better to do many AMPS, because the more one does the easier it gets. Because one has all the criteria in ones head. And one, like now when I've been gone for a while, then it's going to take a longer time. But that doesn't mean that I am going to refrain from it…I'll just have to offset some extra time instead, so that I can do it.

In this way, participants expressed a strong sense of integrity in being able to work according to best practices. However, strategies and priorities in making this work were highly individual for the participants in this study. Moreover, all participants felt that routine was a necessity in making assessments go smoothly.

The daily routine is too busy, there are so many things to learn, before I know it I actually cannot use an assessment. If I don't use it I don't know how to do it anymore, especially AMPS I find difficult so I end up not using it as frequently and in the end I might not be using it at all, sort of my situation now.

Participants expressed a need to create a routine for the use of occupational therapy specific assessments in practice. Moreover, experiences shared here also allude to certain challenges in creating routines, something that is taken up in subsequent themes.

## 6. Simply No Space until After Hours

Among participants, time was experienced as a significant factor impacting upon choices in day-to-day practices as well as the integration of practices to routines. A scarcity in time to complete all expected tasks within their workloads was one aspect in addition to the amount of time it took to perform an assessment such as AMPS, A3, or COPM to name a few. However, these experiences also need to be nuanced.

Well, actually, the staff know about AMPS and A3 and—I'd like to say that I don't have time, but actually if I wanted to make the time I could.

Participants expressed a subtle reflection about the possibility to actually do more if initiative was to be taken. Yet a common experience and sentiment among the participants was that time was a scare commodity. One participant said the following:

So if I do an ADL assessment in preparation for a team planning meeting, so I often do, so I choose Suunnas, because it is quicker. Because if I would do an entire formal AMPS it requires so much more sort of.

Another participant said the following:

And we block, if the patient is in a 4-person room and one chooses to do a shower task and make the bed, well then it takes a long time and then one blocks the bathroom very long.

Participants illustrated in their shared stories about everyday practices that each day was filled with choices based on weighing the pros and cons of choosing a lengthier versus shorter assessment, perhaps based on requiring observations or longer interviews. In the above situation, the participant reflected no ethical contemplation about the choice but rather expressed this as a matter of practicality. Another therapist summarized the sentiment: “I am simply too busy.” However, when therapists felt that they should choose an occupation-based assessment but could not due to time or space, then it introduced a significantly more difficult dilemma. One therapist shared her individual solution to this dilemma:

I have to do evaluations outside of working hours. We only have one small rehab room for staff and clients to share, and in the morning we see about 10 clients and in the afternoon about 7 clients. There is simply no space to do an AMPS evaluation until afterhours.

Strategies such as these can also be seen as reflecting a degree of consideration for peers as well as clients, in avoiding a too great burden. A therapist in another facility responded about how the team solved issues around physical space:

But the more we fix our physical spaces to look like home environments, the easier it gets to do assessments. There are ironing boards, a kitchen, a bathroom, a bed to make and so forth. One works actively here so that patients can access familiar occupations, vacuum and so on.

Similarly, another therapist concurred that environment was an important element in being able to perform assessments such as AMPS.

If I tell the kitchen staff 3 days in advance, um, and oh yeah they ask “what do you need,” for instance how many slices of bread, and they will prepare it, and the hospital covers the costs, and so it is sort of that type of environment, it is easy to manage, it is how it is for me but I realize that I should be thankful for the environment.

All occupational therapists who were interviewed in both Japanese and Swedish contexts concurred that time was scarce in relation to the volume of work that they felt needed to be done. Moreover, the physical environment was also experienced as relevant; however, the environment was to different degrees a barrier or possibility depending on organization.

## 7. Biggest Barriers Can Be the Work Culture

The impact of the work culture on choice of assessments was expressed as a barrier. When participants worked in teams where they experienced consensus and clear leadership, social conformity was in many ways a positive factor. One participant says the following:

One should have consensus, we have two rehabilitation programs and that steers us, because since we work at xx facility, it is a requirement for us to use valid and reliable instruments.

Another participant says the following:

What is there, what we use here is also…it should not just depend on me, what one choses, that one does an assessment.

Surprisingly, however, some therapists expressed that when this sense of consensus works to create a majority culture that does not include the use of client-centered or occupation-based assessments, the greatest challenge is the team itself. One participant says the following:

The biggest barriers are my occupational therapy colleagues. They choose a very biomechanical approach and when their clients see me with my client, they wonder why we are doing things differently and seemingly more fun. Then my colleagues make me feel like I should just do as they do.

The culture of the work environment is vital for the choices made by therapists. In some cases, this culture serves to strengthen a notion of client-centered practice and in other cases it serves to exclude those that try to introduce these ideas.

## 8. Being More Specific: Communication

The positive aspect of working in a client-centered and occupation-based manner was the ability to effectively communicate with clients regarding concrete observations in situations where the client had carried out tasks and where there were concrete understandings about where the person might have done well or was in need of assistance. One participant says the following:

It used to be difficult for me to communicate about performance concretely, but when using AMPS I can be more specific about what the person did (needed help with) and how it specifically related to real examples from daily life.

In this way, the use of AMPS provided an added value that could justify the added time or efforts in performing the assessment.

To be able to communicate is one thing, not only with the family, but with other staff members, it is sort of easier to explain. Since an actual occupation is used, it is clear what aspects the person is able to do or what the person is not able to do and this is directly connected to everyday life. When someone asks me, before it was difficult to be concrete, but with AMPS data I can be very specific about what I saw and how this is related to the goals and plans. I think this is something important.

For participants in this study, using an occupation-based assessment such as AMPS required a time investment for both the client and therapist. However, this process also gave the occupational therapist a greater degree of detail on which to base his/her assessment. This in turn led the participants to raise positive experiences about being able to be more concrete about the results of an assessment with an individual client as well as having a richer context around reported outcomes and intervention goals in a team or patient/family setting.

## 9. Discussion

The main findings from the study highlight what can facilitate and hinder the use of occupational therapy specific assessments in the context of occupational therapy practice in two different country contexts. The findings reflect that incorporating formalized occupational therapy evaluations in practice is challenging on multiple levels. The challenge of integrating new assessments into everyday habits and routines in clinical practice was noteworthy. This is not surprising but it can be concerning that occupational therapists choose assessments based on personal convenience rather than relevance for the case at hand. Given the instruments in focus for recruiting participants to this study, we revisit the Model of Human Occupation.

The Model of Human Occupation (MoHO) [27] can perhaps be useful in informing the lens through which we look when analyzing occupational therapists' experiences. MoHO has been characterized as a body of theory that explains how occupation is motivated, performed, and integrated in daily life. MoHO has primarily been applied to “patient” groups, although by definition applications should be possible to people that for one reason or another experience challenges in performing and/or changing their occupations. Of particular interest, here is the idea of habits and routines. Habitual behavior generally has to do with how people organize occupations in stable and repeatable routines; there is a certain element of automaticity and familiarity which comprises human behavior as habitual within the MoHO. This is of interest because the occupational therapists interviewed in this study raised the dilemma of changing habits in relation to practices in assessment procedures. From the perspective of the human as an occupational being [28, 29], the dilemma raised by the participants in this study can be understood as resistance or as a need to maintain routines in order to be able to work. In the Assessment of Motor and Process Skills (AMPS) manual [2], the need for systematically checking scoring criteria and not relying on habit is desirable for the purposes of validity and reliability of such a standardized evaluation. On the other hand, it is possible as participants in this study purported to establish habits and routines in the use of AMPS, even though the use of AMPS requires the systematic use of a manual. This is a concrete example of the complexity surrounding human occupation when applied to the everyday work of occupational therapists. As illustrated in the findings of this study, on one hand, habit is a necessity for finding a just right challenge and building competencies in performance. On the other hand, habitual action can impact upon the soundness of the results from assessments within occupational therapy practice, thus risking poor outcomes, especially when occupational therapists are implementing changes in their own evaluation practices. Understanding the balance between these two aspects and the need for critical reflection about one's minute practices is of high relevance for evidence-based and person-centered practice.

If occupational therapists choose assessments based more on access than on the immediate needs for the client, there is a risk that the client-centered aspects of practice also are undermined. Shifting power away from the healthcare provider toward the client, prioritizing goals relevant for the client, and focusing on enabling participation in society through occupation are important aspects of client-centeredness here and require the active utilization of relevant assessment tools and intervention models that place the client and his/her needs through occupation into focus. The risk of losing client-centeredness can emerge from small compromises in which a less relevant assessment tool is prioritized because of convenience and in so doing leading to goal setting and intervention planning that neglects empowering the client or focusing on what is most important for him/her [9, 30, 31].

Our findings suggest that the perceptions of the work context largely influenced the choice of a specific assessment. The implementation of new methods is influenced by various social networks. Most of the influencing factors described in our findings are related to individuals or to the organizational environment. Perhaps social networks, when optimal in function, can reduce situations where therapists do not prioritize what they believe is best practice. The participant who said “I'd like to say that I don't have time, but actually if I wanted to make the time I could” reflects something of a dilemma in that the therapist shows a sense of awareness about something lacking in her practice and yet chooses not to prioritize what she considers best practice. Based upon the findings in this study, this phenomenon or trend is something that occupational therapists need to look more carefully at in order to improve possibilities for changing practices and for further work in the integration of research and clinical practice [6, 32]. Although being beyond the scope of this paper, it can also be of interest for occupational therapists to look at implementation science as a way to facilitate the integration of best practices [33].

This study also highlights a discrepancy between how occupational therapists think they should work and how they actually work. The difficulty of incorporating knowledge and changing practice has been reported elsewhere [7, 8, 34, 35]; however, it is interesting to note that incorporating thinking about one's practice seems to be characteristic of therapists who have engaged in continued education [36]. Initially, the authors expected notable differences in experience based on country context; however, the data does not support this assumption. Instead, more generic aspects of practice context and organizational culture appear to have a stronger influence on the implementation process. In order to find innovative strategies to bridge gaps between recommendations and usual practice within clinical settings, it is vital to know what factors impact upon implementation of new methods.

*Limitations*. There are several limitations to this study. It was not possible to conduct data gathering in two country contexts within the same period, which meant that interview guides could not be revised based on two country contexts. On the other hand, the aim of the study was not to compare country contexts but rather to use Sweden and Japan because both were contexts in which many occupational therapists with AMPS training were active and also were in an active process of learning and implementing additional client-centered and occupation-based or occupation-focused evaluations in practice. The two contexts were thought to contribute with different cultural dimensions. Interestingly, the findings would suggest that Swedish and Japanese contexts have less to do with occupational therapy specific assessments than the practice contexts.

A limitation was that only therapists with initial AMPS and A3 training were recruited. Since there is previous research suggesting that it is difficult to implement time-intensive tools such as AMPS in practice, despite extensive training and in fact also suggested in the findings from this study, then it is of relevance to look at occupational therapists that arguably have optimal conditions. Another limitation is that information about why potential participants did not participate is not available. Information about the study was circulated to course participants in the A3 course database. There can be several reasons for why people did not respond, including the fact that email addresses could have changed, lack of interest in the study, or other competing priorities to name a few. It is possible that those who took initiative to participate were particularly interested in actively pursuing the use of AMPS and A3 in their practices.

Another limitation is that some interview questions were formulated, such as “what do you experience as challenges using these assessments?” or “can you describe how these assessments were helpful in practice?” This can be seen as biasing in that the questions assume that something was challenging or helpful. On the other hand, it is natural in both Swedish and Japanese to talk about challenges and possibilities. If questions had only been about challenges, the bias might have been more limiting; however, in this case, the questions get at two sides of a phenomenon.

Finally, it can be a limitation to have combined individual and focus group interviews because the type of data generated is different. However, the benefits of enriching the data by including individual interviews were seen to outweigh the drawbacks. Some participants had little flexibility in their schedules and therefore found it difficult to participate at the set time and place for a focus group. By incorporating individual interviews, the data also reflects voices among those who would not otherwise have been included.

## 10. Conclusion

The aim of this study was to explore occupational therapists' experiences in implementing the AMPS in combination with other client-centered and occupation-based or occupation-focused assessments within various clinical practice settings in Sweden and Japan. In keeping with previous studies, occupational therapists in a myriad of clinical and country contexts find it challenging to implement client-centered and occupation-based assessment tools into practice. More work is needed to understand how best practices can be implemented and sustained through the whole continuum of occupational therapy practice.

## Figures and Tables

**Table 1:** Aggregated demographics.

Demographics	Characteristic	*n* = 19
(i) Sex	Female	14
	Male	5

(ii) Country context	Sweden	7
	Japan	12

(iii) Work experience	General hospital (incl. rehabilitation)	11
	Home visit rehabilitation	1
	Education	3
	Nursing home	2
	Psychiatry facility	2

(iv) Interview style	Individual	8
	Focus group	11

## References

[1] Law M., Baptiste S., McColl M. A., Opzoomer A., Polatajko H., Pollock N. (1990). The Canadian occupational performance measure: an outcome measure for occupational therapy. *Canadian Journal of Occupational Therapy*.

[2] Fisher A. G. (2003). *Assessment of Motor and Process Skills: Development, Standardization and Administration Manual*.

[3] Eriksson G., Tham K., Borg J. (2006). Occupational gaps in everyday life 1–4 years after acquired brain injury. *Journal of Rehabilitation Medicine*.

[4] Fisher A. G., Griswold L. A. (2009). *Evaluation of Social Interaction*.

[5] Fisher A. G. (2013). Occupation-centred, occupation-based, occupation-focused: Same, same or different?. *Scandinavian Journal of Occupational Therapy*.

[6] Sirkka M., Zingmark K., Larsson-Lund M. (2014). A process for developing sustainable evidence-based occupational therapy practice. *Scandinavian Journal of Occupational Therapy*.

[7] Alotaibi N. M., Reed K., Nadar M. S. (2009). Assessments used in occupational therapy practice: an exploratory study. *Occupational Therapy in Health Care*.

[8] Chard G. (2000). An investigation into the use of the Assessment of Motor and Process Skills (AMPS) in clinical practice. *The British Journal of Occupational Therapy*.

[9] Öhman J. I. K., Asaba E. (2014). Goal setting in occupational therapy: a narrative study exploring theory and practice in psychiatry. *World Federation of Occupational Therapists Bulletin*.

[10] Kaneyama T., Inoue K. (2010). A survey of ADL assessment approaches used by occupational therapists in practice. *Okayama Journal of Occupational Therapy*.

[11] Iwama M. (2003). Toward culturally relevant epistemologies in occupational therapy. *American Journal of Occupational Therapy*.

[12] Mattingly C. (1998). In search of the good: Narrative reasoning in clinical practice. *Medical Anthropology Quarterly*.

[13] Mattingly C. (1998). *Healing Dramas and Clinical Plots: The Narrative Structure of Experience*.

[14] Asaba E., Petersson I., Bontje P., Kottorp A. (2012). The assessment of awareness of ability (A3) in a Japanese context: A Rasch model application. *Scandinavian Journal of Occupational Therapy*.

[15] Polkinghorne D. E. (1995). Narrative configuration in qualitative analysis. *International Journal of Qualitative Studies in Education*.

[16] Pope C., Ziebland S., Mays N., Pope C., Mays N. (2006). Analysing qualitative data. *Qualitative Research in Healthcare*.

[17] Asaba E., Asaba A. (2010). Towards client-centered and occupation-based evaluation and intervention: A case study. *Okayama Journal of Occupational Therapy*.

[18] Fisher A. G., Griswold L. A., Munkholm M., Kottorp A. (2017). Evaluating domains of everyday functioning in people with developmental disabilities. *Scandinavian Journal of Occupational Therapy*.

[19] Kottorp A., Heuchemer B., Lie I. P., Gumpert C. H. (2013). Evaluation of activities of daily living ability and awareness among clients in a forensic psychiatry evaluation unit in Sweden. *The British Journal of Occupational Therapy*.

[20] Kottorp A., Ekstam L., Petersson Lie I. (2013). Differences in awareness between persons with left and right hemispheric stroke. *Scandinavian Journal of Occupational Therapy*.

[21] Kottorp A., Petersson I. (2011). Psychometric evaluation of an assessment of awareness using two different Rasch models. *Scandinavian Journal of Occupational Therapy*.

[22] Öhman A., Nygård L., Kottorp A. (2011). The relationship between occupational performance and awareness of disability in older adults with cognitive impairment or dementia. *Scandinavian Journal of Occupational Therapy*.

[23] Anderson R. L., Doble S. E., Merritt B. K., Kottorp A. (2010). Assessment of awareness of disability measures among persons with acquired brain injury. *Canadian Journal of Occupational Therapy*.

[24] Seidman I. (1998). *Interviewing as Qualitative Research: A Guide for Researchers in Education and the Social Sciences*.

[25] Ayres L., Given L. (2008). Thematic coding and analysis. *The SAGE Encyclopedia of Qualitative Research Methods*.

[26] Muhr T. (1997). *ATLAS.ti. WIN 4.2 Build 061*.

[27] Kielhofner G. (2008). *Model of Human Occupation: Theory and Application*.

[28] Wilcock A. (1998). *An Occupational Perspective of Health*.

[29] Cutchin M. P., Aldrich R. M., Bailliard A. L., Coppola S. (2008). Action theories for occupational science: The contributions of Dewey and Bourdieu. *Journal of Occupational Science*.

[30] Maitra K. K., Erway F. (2006). Perception of client-centered practice in occupational therapists and their clients. *American Journal of Occupational Therapy*.

[31] Wressle E., Samuelsson K. (2004). Barriers and bridges to client-centred occupational therapy in Sweden. *Scandinavian Journal of Occupational Therapy*.

[32] Sirkka M., Larsson-Lund M., Zingmark K. (2014). Occupational therapists' experiences of improvement work: A journey towards sustainable evidence-based practice. *Scandinavian Journal of Occupational Therapy*.

[33] May C. R., Johnson M., Finch T. (2016). Implementation, context and complexity. *Implementation Science*.

[34] Upton D., Stephens D., Williams B., Scurlock-Evans L. (2014). Occupational therapists' attitudes, knowledge, and implementation of evidence-based practice: A systematic review of published research. *The British Journal of Occupational Therapy*.

[35] Balas E. A., Boren S. A., Bemmel J., McCray A. T. (2000). Managing clinical knowledge for health care improvement. *Yearbook of Medical Informatics 2000: Patient-Centered Systems*.

[36] Nygård L., Kottorp A., Rosenberg L. (2015). Making use of research: Clinical views on an evaluation of everyday technology use. *Scandinavian Journal of Occupational Therapy*.

